# Microfluidic Technologies for Synthetic Biology

**DOI:** 10.3390/ijms12063576

**Published:** 2011-06-03

**Authors:** Parisutham Vinuselvi, Seongyong Park, Minseok Kim, Jung Min Park, Taesung Kim, Sung Kuk Lee

**Affiliations:** 1 School of Nano-Bioscience and Chemical Engineering, Ulsan National Institute of Science and Technology, 100 Banyeon-ri, Ulsan 689–798, Korea; E-Mails: vinu23pari@unist.ac.kr (P.V.); wjdals24@unist.ac.kr (J.M.P.); 2 School of Mechanical and Advanced Materials Engineering, Ulsan National Institute of Science and Technology, 100 Banyeon-ri, Ulsan 689–798, Korea; E-Mails: sypark0215@unist.ac.kr (S.P.); prodeus1985@unist.ac.kr (M.K.)

**Keywords:** microfluidics, synthetic biology, genetic circuits, gene expression and regulation, metabolite detection, whole-cell analysis

## Abstract

Microfluidic technologies have shown powerful abilities for reducing cost, time, and labor, and at the same time, for increasing accuracy, throughput, and performance in the analysis of biological and biochemical samples compared with the conventional, macroscale instruments. Synthetic biology is an emerging field of biology and has drawn much attraction due to its potential to create novel, functional biological parts and systems for special purposes. Since it is believed that the development of synthetic biology can be accelerated through the use of microfluidic technology, in this review work we focus our discussion on the latest microfluidic technologies that can provide unprecedented means in synthetic biology for dynamic profiling of gene expression/regulation with high resolution, highly sensitive on-chip and off-chip detection of metabolites, and whole-cell analysis.

## 1. Introduction

### 1.1. Synthetic Biology

The development of bioinformatics and functional genomics has enabled not only the ability to understand or modify existing biological systems but also to create new biological systems for special purposes. The natural outcome of such an advance is synthetic biology, which deals with the design and assembly of predictable and robust biological parts/systems and systems biology, which aims at system-level understanding of biological systems. These well-characterized and novel biological parts/systems would in turn provide useful drugs, green fuels, or other high value biomaterials [1,2]. Synthetic biologists differ from genetic engineers in that they try to engineer and create complex biological systems for practical applications from lesser understood and unreliable basic components [3]. Systems biologists develop tools of modeling, simulation, and comparison to experiment in order to understand complex biological systems. The systems biology approach will be especially useful in synthetic biology. Challenges associated with the progress in synthetic biology and systems biology will be the focus of this review.

The two main challenges that limit the progress of synthetic biology are the complexity of the biological systems and the physical variations in biological behavior. These limitations lead to an uncertain probability of success of the engineered biological systems and an inability to fully predict even a simple component [3,4]. Despite several advances in synthetic biology, engineering biological systems is still an expensive, time-consuming, and unreliable process [4]. The response of a biological system is usually nonlinear in that even a simple pathway in a well-studied microorganism cannot be explained satisfactorily. The task of creating an artificial biological system is made more complex with the increase in the complexity of the novel genetic circuits.

Unlike other electrical systems in which interactions between individual components are well characterized and the components operate independently, with the biological components, one cannot avoid undesirable crosstalk due to nonspecific interactions with other components in the cell. Understanding the overall behavior of the natural system is a prerequisite for the successful design of a synthetic biological system. High-throughput experimental methods are necessary to understand the complexity associated with biological systems [5]. Automated, multiplex, and parallel reactions are mandatory in order to gain a deeper insight into the complex biological systems. Microarrays, microplate readers [5], flow cytometers, and fluorescence microscopes [6] are currently being used for high-throughput screening. However, these screening methods are limited by their small sample space, noisy output caused by the spatial proximity of the samples, and the lack of facilities for time-lapse experiments [7]. Time-lapse experiments are particularly important in order to understand the dynamics of gene regulation [6].

### 1.2. Microfluidics

Microfluidics is an analytical system enabling the processing and manipulation of small amounts of fluids. Microfluidic technology has been a significant attraction for biochemists, biologist, analytical chemists, and others as it has demonstrated a capability to reduce cost and labor and also enhance resolution and precision. A single chip enables high-throughput continuous and batch processing of multiple samples both in series and in parallel. Therefore, it is believed that microfluidics can provide unprecedented approaches for synthetic biology. The advantages offered by miniaturization could be exploited to study the complexity associated with biological systems [1]. Microfluidic tools are especially useful in biological studies for analyzing a large number of samples simultaneously and providing dynamic and controlled micro-environmental conditions. Apart from allowing sensitive and robust analysis at lower cost, microfluidics also offers several superior tools to aid the development of synthetic biology. Concurrently, microfluidics has the possibility to resolve the limitations of existing tools for synthetic biology: blending the microfluidics platform with synthetic biology makes it possible to ascertain the dynamics of a gene network in a single cell because the platform provides well separated compartments for single cells with the ability to introduce rapid environmental perturbations [6]. Time-lapse experiments are also made possible with the advent of microfluidics [8]. The cost of microscale multiplex experiments is several fold lesser than that of macroscale multiplexing. Microfluidics can increase the number of samples that can be analyzed: as many as 1.5 million samples can be analyzed simultaneously [9]. Microfluidics forms a common platform for analysis of both bacterial and mammalian cells.

### 1.3. Blending Microfluidics with Synthetic Biology

For synthetic biology to advance further and attain its final goal of a synthetic cell with a desired phenotype, it is necessary to rapidly characterize and understand the dynamics of gene regulation. The controllable environments offered by microfluidic technology can accelerate the process for the achievement of the final goal of synthetic biology. Without genome-wide data on gene dynamics, it is impossible to understand biological complexity. The biological data obtained by conventional macroscale methods appears to be insufficient to completely understand the efficacy of natural biological systems/parts. Many attempts were made to blend microfluidic technology with synthetic biology to multiplex gene synthesis, accelerate DNA sequencing, and analyze the effect of a multifunctional micro-environment on a single cell. Use of microfluidic technology in synthetic biology does not end just with the synthesis or sequencing of biological parts (DNA, in particular) but extends further to favor a deeper understanding of the part in the context of the whole cell [9]. Microfluidic technology has also revolutionized other areas of synthetic biology such as understanding the dynamics of gene regulation, detection of the intra/extracellular metabolites, and whole-cell analysis.

Aided by microfluidic devices, the expression of biological parts in the whole cell can be controlled and regulated and the metabolites produced can be detected by both invasive and noninvasive techniques [1]. Microfluidic devices help in integrating the two major analytical techniques (sampling and assaying) on a single chip, which can reduce the time needed for biological assay and favor real-time monitoring [10]. They also offer room for immobilization and controlled transport of cells. Since microfluidic devices are small, they favor accumulation of nutrients and hence form a stable microenvironment around the cell. Continuous-liquid-flow-type microfluidic devices can be used for long-term culture of cells, as the waste is removed and nutrients are replenished continuously. Optical tweezers facilitate the analysis of a single cell at high resolution [11]. Complex microfluidic devices with an array of cells, each controlled individually by valves, may help perform several parallel experiments [12]. Flow-switching valves can be used to manipulate the environment of the cell with time and hence can help understand the dynamics of gene regulation. Microfluidic devices that can produce a spatial gradient of chemicals can be a tool in understanding the mechanism of chemotaxis and quorum sensing, where the concentration of the signaling molecule determines the fate of the cell [13]. Microfluidics-based *in vitro* compartmentalization and droplet-based microfluidics are highly promising tools for performing parallel reactions. Slipchips are recently emerging as a novel tool showing a high potential for high-throughput parallel screening of various parameters on a sample and for multiplexed applications such as nanoliter PCR arrays on a chip [14,15]. Microfluidic devices coupled with optical tweezers have been designed to perform whole-cell assays and to study the mechanism of chemotaxis in *Escherichia coli* [16,17]. The contribution made by microfluidic technology to the progress of synthetic biology is vast. In this review, we highlight the latest contributions made by microfluidics to the understanding of the dynamics of synthetic bacterial systems.

## 2. Gene Expression and Regulation

Understanding the dynamics of gene expression and regulation forms the foundation of synthetic biology. Upon completion of the construction of a synthetic biological component, the first step is functional assessment of gene expression. It is desirable to analyze the variation in gene expression with respect to different environmental stimuli in order to precisely identify the functions of synthetic parts/systems [18]. Current methods for the assessment of gene expression involve the use of fluorescent protein expression in microplate readers and flow cytometers. However, these assessment tools are still insufficient for screening the rapid response of a cellular system to different environmental stimuli, and the detection limit restricts the analysis to proteins that are highly expressed. Such limitations of current technologies should be resolved, and better methods are required for the development of synthetic biology. However, in microfluidic devices, cells can be confined to a very small space and, hence, the signal from even a small concentration of a protein (in particular, regulatory proteins) is amplified several fold, thus allowing real-time monitoring of the activity of the protein within a cell [19]. Without exploiting the advantage of the concentrator offered by microfluidics, it is almost impossible to determine the effect of regulatory proteins as their expression level is below the detection range of a macroscale device. There are several microfluidic devices for better understanding gene expression and regulation, which are highlighted in the following section. Miniaturized methods to monitor and control gene expression and regulation of synthetic biological parts on a chip can be largely categorized as follows: droplet-based methods for single-cell analysis and array-based method for the analysis of the effect of environmental changes on gene expression.

**Figure 1:**
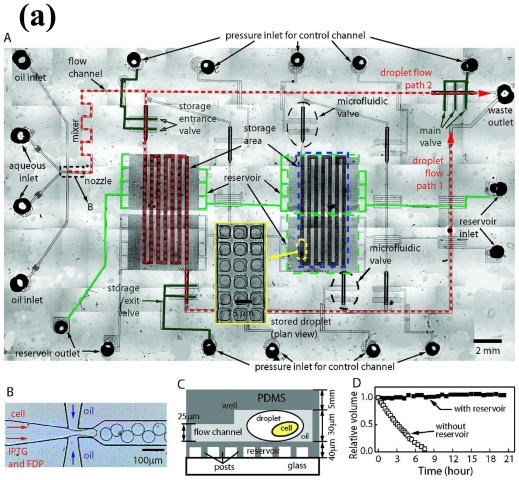

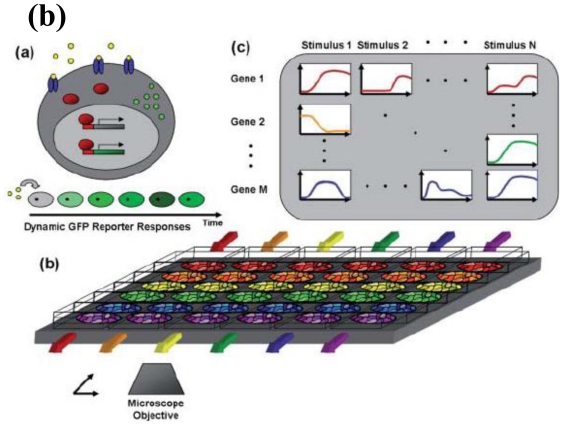
**(a)** Compartment-based microfluidics for simultaneous determination of gene expression and enzyme activity. The image is reproduced with the permission of the Journal of American Chemical Society [23]; **(b)** High-throughput array-based microfluidic device that enables real-time characterization of gene expression. The image is reproduced with the permission of the Royal Society of Chemistry [24].

Droplet-based, quantitative detection of gene expression has been achieved even at the single-cell level [20] and many review and research papers have already highlighted the unique advantages of droplet-based microfluidics for monitoring gene expression [1,21,22]. For example, Huebner *et al.* encapsulated single cells into aqueous microdroplets and then detected the expression of a fluorescent protein individually [15]. Due to the capability for high-throughput analysis (>10^7^ sample throughput per day), droplet-based gene expression analysis can be applied to many biological studies. Also, as shown in Figure 1(a), Shim *et al.* demonstrated the compartmentalization of single bacterial cell within a droplet of picoliter volume on a chip [23]. The chip not only facilitated the study of the dynamics of protein expression but also measured enzymatic activity in individual cells. This can be a powerful tool for investigating the heterogeneity of cells in identical culture environments. However, some of the bottleneck issues related to droplet-based microfluidics include droplet shrinkage, size variations, encapsulation of cells based on poisson distribution and intra-group variations. In addition to droplet-based methods, microfluidic-array-based high-throughput devices have been developed [24–27]. In particular, Thompson *et al*. reported a microfluidic array device for high-throughput analysis of gene expression profiles using the phenomenon of diffusive mixing in a cell culture chamber [25]. King *et al*. developed a similar array-based high-throughput microfluidic device capable of analyzing gene expression in living cells and revealed a distinct dynamics in gene expression (Figure 1(b)) [24].

Regulation of gene expression in response to both intracellular and extracellular stimuli can be analyzed using microfluidic devices. Methods to introduce intracellular stimuli require highly elaborate microdevices or functional nanoparticles to access the insides of the cell. Recently, nanoparticles have been widely developed as a novel means for applying intracellular stimuli to regulate gene expression, but this is beyond the scope of our review [28]. Instead, extracellular methods are used, which typically involve the application of mechanical or chemical stimuli to regulate gene expression in the cells [29]. Microfabricated and mechanically confined microenvironments have been used to investigate the persistence of antibiotic resistance in *E. coli* by allowing cells to grow and divide in straight microchannels [30]. Using these mechanical confinement interfaces, Balaban *et al*. revealed the phenotypic switching that occurs between cells in the absence of antibiotics (with normal growth rates) and that in the presence of antibiotics (with reduced growth rates), and thus was able to relate the inherent heterogeneity in a bacterial cell population to persistence. In addition, microfluidic confinement of single cells is used to study the behavior of quorum sensing on growth, as microfluidic confinement allows us to control and monitor gene expression in a single cell [31]. Temperature gradients in a microfluidic device have been developed using a typical Y-shaped channel [32] and time-specific switching of temperature is used to investigate the patterning of cells [33]. Temperature gradient generator devices can be considered useful for controlling and understanding the effects of extracellular stimuli on gene expression and regulation. Furthermore, chemical stimuli with spatiotemporal gradients have been very widely used to actively regulate gene expression. Many microfluidic devices with biochemical interfaces have been developed that use gradient flow to regulate gene induction or inhibition [34–36]. Charvin *et al*. reported that a microfluidic device can control gene expression temporally and monitored the long-term fluorescence response [37]. Lastly, since oxygen plays a crucial role in regulating cells, a microfluidic oxygen gradient generator device has been developed by using arrays of electrodes transducing current into oxygen via electrolysis [38].

## 3. Metabolite Analysis

In contrast to genomics and proteomics, metabolomics helps explain actual cellular behavior and hence is gaining increased importance in biological studies [39]. By investigating the metabolite, a biologist can analyze an organism’s phenotype faster. For the synthetic biologist, metabolomics is especially crucial in predicting the product yield of an engineered metabolic network. However, decoding the metabolome is very difficult because, with the macro-scale devices, there are no tools to amplify the signal from metabolites that are at submicromolar concentration. The cell extracts usually contain various metabolites in diverse ratios. For that reason, high-resolution separation and sensitive detection are required for metabolite analysis [40]. Tools for metabolite analysis include nuclear magnetic resonance (NMR) spectroscopy, gas chromatography coupled to mass spectrometry (GC-MS), and liquid chromatography coupled to MS (LC-MS) [41]. These instruments favored the development of a database of metabolites and enable label-free, multiple-compound detection. However, the cost, handling, and maintenance of the instruments limit their applications [9] and a time consuming sample preparation step is always needed to guarantee the sensitivity of the techniques. Microfluidics makes new ways for the study of metabolites. Microfluidics can offer a platform for faster sample preparation, better separation, and robust analysis. However, faster analysis does not normally promise a better detection [40]. Extensive microfluidics-based metabolite detection methods have been reviewed elsewhere [40]. In this section, the possibility of monitoring a metabolite (both intracellular and extracellular) on a microfluidic chip is discussed.

### 3.1. Off-Chip Detection

The macroscale metabolite detection techniques demand extensive sample preparation for more accurate analysis. In cases where the analyte concentration is very small, the sample preparation is more tedious and requires skilled workers. Hence, a microfluidic device is used to prepare the metabolite samples (including extraction, concentration and separation) for MS and other macroscale devices. With this method, even the metabolites present at sub-micromolar quantity are sufficiently concentrated for further analysis with the macroscale device connected to the microfluidic chip. The matrix-assisted laser desorption/ionization MS (MALDI-MS) technique was successfully integrated with the dispensing system imprinted with yeast cells and 5–12 attomoles of some important metabolites were detected [42]. More recently, Fidalgo *et al*. combined droplet-based microfluidic technology with fluorescence detection and electron spray ionization-MS (ESI-MS) to isolate droplets containing angiotensin, fluorescently labeled angiotensin, and bradykinin [43]. Gao *et al.* developed a device that interconnected both cell culture and on-chip solid-phase extraction (SPE), leading to the detection of vitamin E produced from human lung epithelial A549 cell lines [44]. Lin *et al*. further linked a microfluidic chip with liquid chromatography (LC), MS, and NMR by connecting a nanosplitter [45]. A fully automated microfluidics-based electroporator was used to separate the proteins of the cell wall of different species of lactic acid bacteria and thus helped resolve the identity of strains at the species level from proteomic data [46]. These approaches took advantage of conventional methods as well as microfluidic technology.

### 3.2. On-Chip Detection

**Figure 2:**
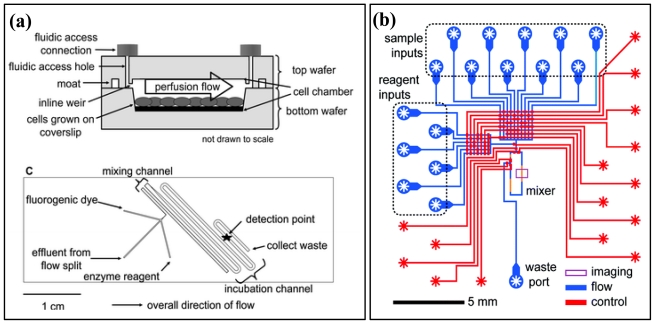
**(a)** Enzyme-based on-chip, *in situ* metabolite monitoring device. Cell culture chip (top) and enzyme assay chip are linked and enable continuous monitoring. The image is reproduced with the permission of Analytical Chemistry [49]. **(b)** Multilayered, autonomous, enzyme-based microfluidic metabolite detection device. Sample preparation, reagent mixing, and data acquisition can be performed without operator intervention. The image is reproduced with the permission of Analytical Chemistry [50].

On-chip detection methods can be categorized into two areas according to the detection method: electrochemical detection and enzymatic detection. Cheng *et al.* proposed the electrochemical detection method on a microchip to measure extracellular pH and intracellular Ca^2+^ concentration in heart cells [47]. Liu *et al.* introduced a capillary electrophoresis (CE) coupled bioluminescence detection method to measure the concentration of cellular ATP in *E. coli* [48]. They used electro-osmotic flow (EOF) and reversed EOF for separating various metabolites in solution. After separation, they mixed enzymes to detect ATP and ATP-conjugated metabolites such as galactose. Davidsson *et al*. developed a microfluidic flow injector (μFIA) for enzymatic detection of glucose and ethanol produced by yeast [2]. They showed that the device could monitor production of glucose and ethanol in yeast in a noninvasive way by linking silicon chips with a fluid line. Clark *et al.* measured glycerol production by adipocytes on a microfluidic enzyme assay chip and achieved a 4 μM detection limit, as shown in Figure 2(a) [49]. They combined a cell culture chip and an enzyme assay chip via a capillary and monitored glycerol production. Urbanski *et al.* developed a noninvasive metabolic profiling system based on a combination of multilayer lithography and enzymatic assay on a chip (Figure 2(b)) [50]. This fully automated fluidic system helped monitor the change in metabolism in a single murine embryo. Huebner *et al.* demonstrated enzyme assay using *E. coli* encapsulated in microdroplets [51]. They tested the difference in alkaline phosphatase activity between normal *E. coli* (BL21) and a mutant (R166S). This method can be used for screening a library of useful mutant cells. However, whole-cell metabolite detection methods in microfluidics should be improved further to efficiently analyze target metabolites amidst a noisy background of biological samples, which require various pretreatments such as cleanup and concentration [40,52,53].

## 4. Whole-Cell Analysis

Another main emphasis of synthetic biology is to understand symbiosis in microbial communities, which works efficiently in multi-cellular environments to perform complex tasks like cellulose degradation, methanogenesis, nitrogen fixation, and degradation of toxic compounds. It is important to understand natural cell communities before developing an artificial cellular community (e.g., quorum sensing systems). Microfluidics not only offers a way for co-culture of several species of bacteria but also provides a platform for single-cell culture. Although bacteria live in symbiosis with other microbes in nature, co-culture of microbes in the laboratory had always been a difficult task due to the competition and dominance between different groups of microbes. Microscale spatial separation of different species of microbes provided with chemical communication helped in the co-culture of different microbial species [54]. Controlled co-culture of a microbial community may help understand and harness beneficial natural microbial communities to create a synthetic community with novel function.

The uncultivable microbial species area major challenge in microbiology. Despite the presence of a large pool of microorganisms that grow in the laboratory, a vast majority of the microorganisms are uncultivable even with a rich medium. The group of uncultivable microbes is of particular interest to the synthetic biologist as they may provide evidence for evolution and are the main source of novel genes. Isolation of a pure culture of uncultivable microbes is impossible without isolation chip (Ichip)-based microfluidics. An Ichip offers a miniaturized diffusion chamber that helped isolate a significant and novel group of microorganisms from environmental samples. The microbial species presented in the Ichip were different from those obtained with a rich medium in a Petri dish [55].

Separation and screening of living cells is an essential preparatory step in not only cell-based biological and physiological studies, but also practical applications such as cell engineering, clinical immunoassay, and drug tests. Whole-cell assay is a pre-requisite in toxicogenomics to study the biological impact of toxic compounds. However, conventional macroscale separation methods in biology, such as sieve filtration and gradient centrifugation, have a high risk of causing damage to the cells due to the strong mechanical stress caused by the ultra-fast rotating speed or the viscous forces generated by micropores on the membranes, and the separation favors collection of cells of similar size rather than individual cells. Also, more elaborate methods have been developed, such as fluorescence-activated cell sorting (FACS) and magnetically activated cell sorting (MACS), that require a large volume of samples and are also labor and time intensive [56]. For miniaturized FACS, Y-shaped junctions are widely utilized for positioning individual cells at the center of a laminar flow controlled by optical tweezers. After detecting a fluorescence signal [57], an EOF [58] can be implemented to switch the position of the cell from the center to one of the edges based on the fluorescence signal. The main advantage of the microfluidic FACS, compared with the conventional FACS, is the ability to sort cells at a faster rate (~100 cells/s) [57]. Also, microfluidic approaches are free from contamination with cells of the previous run as these microchips are disposable, being fabricated from cost-effective materials. In a similar manner, for miniaturized MACS, sample cells are labeled using magnetic beads with an antibody acting as an anchor between the magnetic beads and the cells [59–61]. Then, magnetic fields are induced to control the position of the cells for continuous separation and sorting. Compared with a fluorescence signal, magnetic fields can extend over longer distances and manipulate cells simultaneously, resulting in higher throughput (1011 cells in 30 min) [62].

Active sorting mechanisms rely on external forces such as an optical, magnetic, dielectrophoretic, or acoustic force. However, another class of efficient and continuous cell sorting devices was developed by using passive sorting mechanisms. These mechanisms were combined with several mechanical and physical properties of cells, such as size, density, shape, deformability, and polarizability. The cell separation using the micropillar structure [63,64] and obstacle structures [65,66] are fabricated in microfluidic channels, depend on size and deformability of cells. This approach does not require an additional driving force besides a hydrodynamic flow. Hence, it offers a simple and continuous way to separate any particle or cell on the basis of size [67]. Due to superior abilities of microfluidic technology in the control of the environment surrounding a cell, it is possible to cultivate cells in controllable conditions such as chemical concentration, ambient temperature, and external forces for a longer duration. Controlling chemical concentration gradient and other environmental conditions provides a high possibility for cell separation based on the native and robust chemotactic [17,68], chemostatic [69], and quorum sensing responses of the cell [31]. This approach inherently has no physical or mechanical stress that can cause cell death or mutation. Also, this technique can be utilized to find a unique cell type that has optimized resistance and survival abilities in a certain environment.

## 5. Future Perspectives

It is evident from the above discussion that the main bottleneck in the progress of synthetic biology is the need to completely understand biological systems. Despite the many advances in the technology to assess a biological system, it is very difficult to understand their behavior because of the asynchronous nature exhibited even in a homogenous population of cells. Recently, microfluidics technology has been proving its potential to offer a means to detect biological response at the single-cell level by automation and multiplexing. However, microfluidics technology should be made simpler so that even non-experts can work with it. Microfluidic devices should form a part of the laboratory, like other devices in the lab (for example, PCR machine). Microfluidics offers individual platforms for different fields of biology, such as DNA synthesis, sequencing, DNA amplification, cell free-protein expression and functional genomics, proteomics, and metabolomics. A combination of all these devices would be a robust and time-saving platform for diagnosis of various diseases. Functional screening of drugs with better efficacy would be made simpler with the aid of microfluidics.

The field of synthetic biology has been progressing rapidly with the success of rebooting life from a chemically synthesized genome [71] and multiplex automated genome engineering (MAGE) for large-scale programmed evolution of cells in a week’s time for an improved phenotype [72]. With the robust and advanced analysis methods contributed by microfluidics technology, it will be possible to better understand and analyze biological systems and hence engineer them efficiently. Microfluidic devices can help in screening population of millions of organisms to find the most efficient producer of target enzyme or a fuel. Microfluidics technology will provide the hope that the synthetic biologists dream of constructing and understanding the machinery of life (like engineers control mechanical devices) is not too far from reality.

## 6. Conclusions

Recent progress in genetic engineering and molecular biology has opened a new era of science called synthetic biology. The study intends to build artificial organisms by applying the engineering approach to biology [73]. Synthetic biology is considered to have a huge impact in various industrial fields by providing cheap and readily accessible drugs, developing new anti-cancer drugs, and producing biofuels. One excellent example is artificial artemisinin, an antimalarial drug produced by engineered *E. coli* [2]. The synthetic biologists deal with biological parts (modules, circuits, and systems), similar to modern electronic engineers in many ways. In electronics, engineers design circuits using quantitative knowledge of device function; however, the synthetic biologist designs a synthetic organism using a quantitative approach for the genes and biological pathways. While electronics perfectly controls signal transmission by restricting the signal line, the biological counterparts are disturbed by multiple bypass pathways due to the stochastic behavior of each component [74]. To solve these problems, new techniques that can offer stable and robust tools for precise control of microenvironments and reactions on the cellular level are highly required. Thus, microfluidic techniques are a vital and key technology in synthetic biology [9]. The advantages of microfluidics are obvious. Compared with typical pipette-based lab-scale equipments that deal with milliliter to microliter volumes of fluid, the microfluidic technique deals with just nanoliter to picoliter volumes and hence requires lesser reagents. In addition, flow in a microchannel is laminar rather than turbulent due to the size of the microchannel (typically, a few micrometers) and thus favors a highly predictable and controllable flow. Thanks to the recent developments in microfabrication technology, physical and geographical interactions between cells and environments can be studied in ways that were previously not possible with conventional technologies. With these advantages, microfluidics technology revolutionizes the way we study cellular environments. The technology has been successfully applied to many biological problems, especially high-speed PCR [75,76], gene sequencing [77,78], high-throughput screening [79–81], and quantitative analysis of multiple or single cells [82–84]. The technologies used for monitoring synthetic bacterial cell-to-cell signaling [85,86], screening of biomass-to-biofuel conversion enzyme [87,88], and *in situ* monitoring of synthetic organisms [30,69,89] have shown great promise with the combination of these two fields.

A paradigm shift from macroscale methods to microscale analysis of biological parts has been a boon for synthetic biology. Microfluidics is one of the best ways to accomplish automation and multiplexing of biological parts. The importance of microfluidics in synthetic biology will be more appreciated if the process of handling the microchips is simplified in order that it could even be managed by a non-expert.

## Figures and Tables

**Figure 3:**
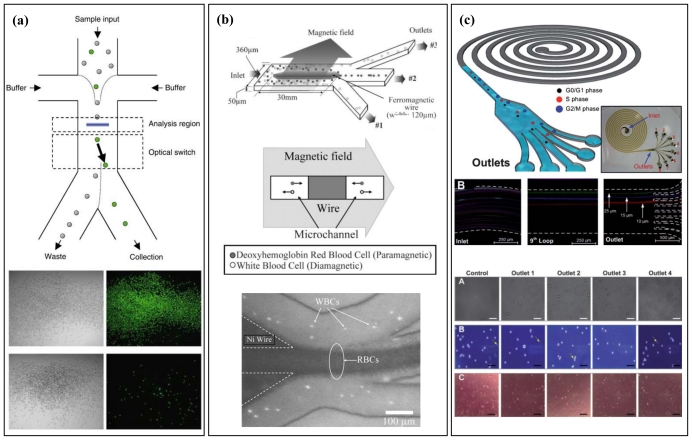
**(a)** Miniaturized FACS. Cells are analyzed and then sorted on the basis of the detected fluorescence signals. Target cells are directed by the laser to the collection output, whereas all other cells flow to the waste output. The image is reproduced with the permission of Nature Biotechnology [58]. **(b)** Miniaturized MACS that contain a patterned ferromagnetic wire in the microchannel. Under a magnetic field across the microchannel, paramagnetic-labeled RBCs come close to the central wire, whereas diamagnetic-labeled WBCs experience repulsion from the central wire. The image is reproduced with the permission of the Royal Society of Chemistry [59]. **(c)** Under the influence of inertial lift forces and Dean drag forces, asynchronous cell populations are size-fractionated to obtain relatively pure populations of cells. The image is reproduced with the permission of the Royal Society of Chemistry [70].

**Table 1:** Microfluidics for advancing synthetic biology.

Microfluidic Device	Potential Application in Synthetic Biology
Device with array of cells	Parallel reaction, gene expression analysis at the single-cell level
Device with switchable valves	The study of dynamics of gene regulation, automation
Chemical concentration gradient generators	Chemotaxis analysis, quorum sensing analysis, toxicity analysis
Microfluidic bioreactor	Evolutionary adaptation through long-term culture, multiplexing, bacterial growth, quantification of bacterial cells
Droplet-based microfluidics	Spatially separated parallel reaction, multiplexing, functionbased high-throughput screening of engineered enzymes
*In vitro* compartmentalization	Parallel reaction, analysis of bacterial community structure, synthetic consortium analysis

## References

[1] Gulati S, Rouilly V, Niu X, Chappell J, Kitney RI, Edel JB, Freemont PS, de Mello AJ (2009). Opportunities for microfluidic technologies in synthetic biology. J. R. Soc. Interface.

[2] Khalil AS, Collins JJ (2010). Synthetic biology: Applications come of age. Nat. Rev. Genet.

[3] Andrianantoandro E, Basu S, Karig DK, Weiss R (2006). Synthetic biology: New engineering rules for an emerging discipline. Mol Syst Biol.

[4] Endy D (2005). Foundations for engineering biology. Nature.

[5] Breslauer DN, Lee PJ, Lee LP (2006). Microfluidics-based systems biology. Mol. Biosyst.

[6] Bennett MR, Hasty J (2009). Microfluidic devices for measuring gene network dynamics in single cells. Nat. Rev. Genet.

[7] Elowitz MB, Levine AJ, Siggia ED, Swain PS (2002). Stochastic gene expression in a single cell. Science.

[8] Locke JCW, Elowitz MB (2009). Using movies to analyse gene circuit dynamics in single cells. Nat. Rev. Micro.

[9] Szita N, Polizzi K, Jaccard N, Baganz F (2010). Microfluidic approaches for systems and synthetic biology. Curr. Opin. Biotechnol.

[10] Yi C, Li C-W, Ji S, Yang M (2006). Microfluidics technology for manipulation and analysis of biological cells. Anal. Chim. Acta.

[11] Ozkan M, Wang M, Ozkan C, Flynn R, Esener S (2003). Optical manipulation of objects and biological cells in microfluidic devices. Biomed. Microdevices.

[12] Anderson JR, Chiu DT, Jackman RJ, Cherniavskaya O, McDonald JC, Wu H, Whitesides SH, Whitesides GM (2000). Fabrication of topologically complex three-dimensional microfluidic systems in PDMS by rapid prototyping. Anal. Chem.

[13] Jeon NL, Dertinger SKW, Chiu DT, Choi IS, Stroock AD, Whitesides GM (2000). Generation of solution and surface gradients using microfluidic systems. Langmuir.

[14] Li L, Du W, Ismagilov RF (2009). Multiparameter screening on slipchip used for nanoliter protein crystallization combining free interface diffusion and microbatch methods. J. Am. Chem. Soc.

[15] Shen F, Du W, Davydova EK, Karymov MA, Pandey J, Ismagilov RF (2010). Nanoliter multiplex PCR arrays on a slipchip. Anal. Chem.

[16] Wakamoto Y, Umehara S, Matsumura K, Inoue I, Yasuda K (2003). Development of non-destructive, non-contact single-cell based differential cell assay using on-chip microcultivation and optical tweezers. Sens. Actuators B.

[17] Mao H, Cremer PS, Manson MD (2003). A sensitive, versatile microfluidic assay for bacterial chemotaxis. Proc. Natl. Acad. Sci. USA.

[18] Eric Y, Hal A (2010). Synthetic biology: Tools to design, build, and optimize cellular processes. J. Biomed. Biotech.

[19] Davidsson R, Johansson B, Passoth V, Bengtsson M, Laurell T, Emneus J (2004). Microfluidic biosensing systems Part II. Monitoring the dynamic production of glucose and ethanol from microchip-immobilised yeast cells using enzymatic chemiluminescent μ-biosensors. Lab Chip.

[20] Huebner A, Srisa-Art M, Holt D, Abell C, Hollfelder F, de Mello AJ, Edel JB (2007). Quantitative detection of protein expression in single cells using droplet microfluidics. Chem Commun.

[21] Vyawahare S, Griffiths AD, Merten CA (2010). Miniaturization and parallelization of biological and chemical assays in microfluidic devices. Chem. Biol.

[22] Casadevall i Solvas X, deMello A (2011). Droplet microfluidics: Recent developments and future applications. Chem. Commun.

[23] Shim JU, Olguin LF, Whyte G, Scott D, Babtie A, Abell C, Huck WTS, Hollfelder F (2009). Simultaneous determination of gene expression and enzymatic activity in individual bacterial cells in microdroplet compartments. J. Am. Chem. Soc.

[24] King KR, Wang SH, Irimia D, Jayaraman A, Toner M, Yarmush ML (2007). A high-throughput microfluidic real-time gene expression living cell array. Lab Chip.

[25] Thompson DM, King KR, Wieder KJ, Toner M, Yarmush ML, Jayaraman A (2004). Dynamic gene expression profiling using a microfabricated living cell array. Anal. Chem.

[26] Ingham CJ, Sprenkels A, Bomer J, Molenaar D, van den Berg A, Vlieg JETV, de Vos WM (2007). The micro-petri dish, a million-well growth chip for the culture and high-throughput screening of microorganisms. Proc. Natl. Acad. Sci. USA.

[27] Ryley J, Pereira-Smith OM (2006). Microfluidics device for single cell gene expression analysis in *Saccharomyces cerevisiae*. Yeast.

[28] Jiang W, Kim BYS, Rutka JT, Chan WCW (2008). Nanoparticle-mediated cellular response is size-dependent. Nat. Nanotech.

[29] Cho YK, Shin H, Lee SK, Kim T (2010). Current application of micro/nano-interfaces to stimulate and analyze cellular responses. Ann. Biomed. Eng.

[30] Balaban NQ, Merrin J, Chait R, Kowalik L, Leibler S (2004). Bacterial persistence as a phenotypic switch. Science.

[31] Boedicker JQ, Vincent ME, Ismagilov RF (2009). Microfluidic confinement of single cells of bacteria in small volumes initiates high-density behavior of quorum sensing and growth and reveals its variability. Angew. Chem. Int. Ed.

[32] Hatch A, Kamholz AE, Hawkins KR, Munson MS, Schilling EA, Weigl BH, Yager P (2001). A rapid diffusion immunoassay in a T-sensor. Nat. Biotech.

[33] Frasch M, Hoey T, Rushlow C, Doyle H, Levine M (1987). Characterization and localization of the even-skipped protein of Drosophila. EMBO J.

[34] Ashe HL, Briscoe J (2006). The interpretation of morphogen gradients. Development.

[35] Lander AD (2007). Morpheus unbound: Reimagining the morphogen gradient. Cell.

[36] Gurdon JB, Bourillot PY (2001). Morphogen gradient interpretation. Nature.

[37] Charvin G, Cross FR, Siggia ED (2008). A microfluidic device for temporally controlled gene expression and long-term fluorescent imaging in unperturbed dividing yeast cells. PLoS ONE.

[38] Park J, Bansal T, Pinelis M, Maharbiz MM (2006). A microsystem for sensing and patterning oxidative microgradients during cell culture. Lab Chip.

[39] Blow N (2008). Biochemistry’s new look. Nature.

[40] Kraly JR, Holcomb RE, Guan Q, Henry CS (2009). Review: Microfluidic applications in metabolomics and metabolic profiling. Anal. Chem. Acta.

[41] Theodoridis G, Gika HG, Wilson ID (2008). LC-MS-based methodology for global metabolite profiling in metabonomics/metabolomics. Trends Anal. Chem.

[42] Amantonico A, Oh JY, Sobek J, Heinemann M, Zenobi R (2008). Mass spectrometric method for analyzing metabolites in yeast with single cell sensitivity. Angew. Chem. Int. Ed.

[43] Fidalgo LM, Whyte G, Ruotolo BT, Benesch JLP, Stengel F, Abell C, Robinson CV, Huck WTS (2009). Coupling microdroplet microreactors with mass spectrometry: Reading the contents of single droplets online. Angew. Chem. Int. Ed.

[44] Gao D, Wei HB, Guo GS, Lin JM (2010). Microfluidic cell culture and metabolism detection with electrospray ionization quadrupole time-of-flight mass spectrometer. Anal. Chem.

[45] Lin YQ, Schiavo S, Orjala J, Vouros P, Kautz R (2008). Microscale LC-MS-NMR platform applied to the identification of active cyanobacterial metabolites. Anal. Chem.

[46] Lonigro SL, Valerio F, de Angelis M, de Bellis P, Lavermicocca P (2009). Microfluidic technology applied to cell-wall protein analysis of olive related lactic acid bacteria. Int. J. Food Microbiol.

[47] Cheng W, Klauke N, Sedgwick H, Smith GL, Cooper JM (2006). Metabolic monitoring of the electrically stimulated single heart cell within a microfluidic platform. Lab Chip.

[48] Liu BF, Ozaki M, Hisamoto H, Luo QM, Utsumi Y, Hattori T, Terabe S (2005). Microfluidic chip toward cellular ATP and ATP-conjugated metabolic analysis with bioluminescence detection. Anal. Chem.

[49] Clark AM, Sousa KM, Jennings C, MacDougald OA, Kennedy RT (2009). Continuous-flow enzyme assay on a microfluidic chip for monitoring glycerol secretion from cultured adipocytes. Anal. Chem.

[50] Urbanski JP, Johnson MT, Craig DD, Potter DL, Gardner DK, Thorsen T (2008). Noninvasive metabolic profiling using microfluidics for analysis of single preimplantation embryos. Anal. Chem.

[51] Huebner A, Olguin LF, Bratton D, Whyte G, Huck WTS, de Mello AJ, Edel JB, Abell C, Hollfelder F (2008). Development of quantitative cell-based enzyme assays in microdroplets. Anal. Chem.

[52] Svobodova J, Mathur S, Muck A, Letzel T, Svatos A (2010). Microchip-ESI-MS determination of dissociation constant of the lysozyme-NAG(3) complex. Electrophoresis.

[53] Reichmuth DS, Shepodd TJ, Kirby BJ (2005). Microchip HPLC of peptides and proteins. Anal. Chem.

[54] Kim HJ, Boedicker JQ, Choi JW, Ismagilov RF (2008). Defined spatial structure stabilizes a synthetic multispecies bacterial community. Proc. Natl. Acad. Sci. USA.

[55] Nichols D, Cahoon N, Trakhtenberg EM, Pham L, Mehta A, Belanger A, Kanigan T, Lewis K, Epstein SS (2010). Use of Ichip for high-throughput in situ cultivation of “uncultivable” microbial species. Appl. Environ. Microbiol.

[56] Ashcroft RG, Lopez PA (2000). Commercial high speed machines open new opportunities in high throughput flow cytometry (HTFC). J. Immunol. Methods.

[57] Wang MM, Tu E, Raymond DE, Yang JM, Zhang HC, Hagen N, Dees B, Mercer EM, Forster AH, Kariv I, Marchand PJ, Butler WF (2005). Microfluidic sorting of mammalian cells by optical force switching. Nat. Biotech.

[58] Fu AY, Spence C, Scherer A, Arnold FH, Quake SR (1999). A microfabricated fluorescence-activated cell sorter. Nat. Biotech.

[59] Han KH, Frazier AB (2006). Paramagnetic capture mode magnetophoretic microseparator for high efficiency blood cell separations. Lab Chip.

[60] Inglis DW, Riehn R, Austin RH, Sturm JC (2004). Continuous microfluidic immunomagnetic cell separation. Appl. Phys. Lett.

[61] Pamme N, Wilhelm C (2006). Continuous sorting of magnetic cells via on-chip free-flow magnetophoresis. Lab Chip.

[62] Thiel A, Scheffold A, Radbruch A (1998). Immunomagnetic cell sorting-pushing the limits. Immunotechnology.

[63] Davis JA, Inglis DW, Morton KJ, Lawrence DA, Huang LR, Chou SY, Sturm JC, Austin RH (2006). Deterministic hydrodynamics: Taking blood apart. Proc. Natl. Acad. Sci. USA.

[64] Sethu P, Sin A, Toner M (2006). Microfluidic diffusive filter for apheresis (leukapheresis). Lab Chip.

[65] Choi S, Song S, Choi C, Park JK (2007). Continuous blood cell separation by hydrophoretic filtration. Lab Chip.

[66] Choi S, Song S, Choi C, Park JK (2009). Microfluidic self-sorting of mammalian cells to achieve cell cycle synchrony by hydrophoresis. Anal. Chem.

[67] Huang LR, Cox EC, Austin RH, Sturm JC (2004). Continuous particle separation through deterministic lateral displacement. Science.

[68] Kim M, Kim T (2010). Diffusion-based and long-range concentration gradients of multiple chemicals for bacterial chemotaxis assays. Anal. Chem.

[69] Groisman A, Lobo C, Cho HJ, Campbell JK, Dufour YS, Stevens AM, Levchenko A (2005). A microfluidic chemostat for experiments with bacterial and yeast cells. Nat. Methods.

[70] Lee WC, Bhagat AAS, Huang S, Vliet KJV, Han J, Lim CT (2007). High-throughput cell cycle synchronization using inertial forces in spiral microchannels. Lab Chip.

[71] Gibson DG, Glass JI, Lartigue C, Noskov VN, Chuang RY, Algire MA, Benders GA, Montague MG, Ma L, Moodie MM, Merryman C, Vashee S, Krishnakumar R, Assad-Garcia N, Andrews-Pfannkoch C, Denisova EA, Young L, Qi ZQ, Segall-Shapiro TH, Calvey CH, Parmar PP, Hutchison CA, Smith HO, Venter JC (2010). Creation of a bacterial cell controlled by a chemically synthesized genome. Science.

[72] Wang HH, Isaacs FJ, Carr PA, Sun ZZ, Xu G, Forest CR, Church GM (2009). Programming cells by multiplex genome engineering and accelerated evolution. Nature.

[73] Haseloff J, Ajioka J (2009). Synthetic biology: History, challenges and prospects. J. R. Soc. Interface.

[74] Morange M (2009). A new revolution? The place of systems biology and synthetic biology in the history of biology. EMBO Rep.

[75] Khandurina J, McKnight TE, Jacobson SC, Waters LC, Foote RS, Ramsey JM (2000). Integrated system for rapid PCR-based DNA analysis in microfluidic devices. Anal. Chem.

[76] Spurgeon SL, Jones RC, Ramakrishnan R (2008). High throughput gene expression measurement with real time PCR in a microfluidic dynamic array. PLoS ONE.

[77] Paegel BM, Blazej RG, Mathies RA (2003). Microfluidic devices for DNA sequencing: Sample preparation and electrophoretic analysis. Curr. Opin. Biotechnol.

[78] Bau S, Schracke N, Kranzle M, Wu H, Stahler P, Hoheisel J, Beier M, Summerer D (2009). Targeted next-generation sequencing by specific capture of multiple genomic loci using low-volume microfluidic DNA arrays. Anal. Bioanal. Chem.

[79] Brouzes E, Medkova M, Savenelli N, Marran D, Twardowski M, Hutchison JB, Rothberg JM, Link DR, Perrimon N, Samuels ML (2009). Droplet microfluidic technology for single-cell high-throughput screening. Proc. Natl. Acad. Sci. USA.

[80] Churski K, Korczyk P, Garstecki P (2010). High-throughput automated droplet microfluidic system for screening of reaction conditions. Lab Chip.

[81] Upadhyaya S, Selvaganapathy PR (2010). Microfluidic devices for cell based high throughput screening. Lab Chip.

[82] Villa-Diaz LG, Torisawa YS, Uchida T, Ding J, Nogueira-De-Souza NC, O’Shea KS, Takayama S, Smith GD (2009). Microfluidic culture of single human embryonic stem cell colonies. Lab Chip.

[83] Takayama S (2010). Microfluidic engineering of stem cell niches and 3D tissue models. In Vitro Cell Dev. Biol. Anim.

[84] Wang Y, Chen ZZ, Li QL (2010). Microfluidic techniques for dynamic single-cell analysis. Microchim. Acta.

[85] Choi WS, Ha D, Park S, Kim T (2011). Synthetic multicellular cell-to-cell communication in inkjet printed bacterial cell systems. Biomaterials.

[86] Lee SH, Heinz AJ, Shin S, Jung YG, Choi SE, Park W, Roe JH, Kwon S (2010). Capillary based patterning of cellular communities in laterally open channels. Anal. Chem.

[87] Bharadwaj R, Wong A, Knierim B, Singh S, Holmes BM, Auer M, Simmons BA, Adams PD, Singh AK (2011). High-throughput enzymatic hydrolysis of lignocellulosic biomass via *in-situ* regeneration. Bioresour. Technol.

[88] Bharadwaj R, Chen ZW, Datta S, Holmes BM, Sapra R, Simmons BA, Adams PD, Singh AK (2010). Microfluidic glycosyl hydrolase screening for biomass-to-biofuel conversion. Anal. Chem.

[89] Balagadde FK, You LC, Hansen CL, Arnold FH, Quake SR (2005). Long-term monitoring of bacteria undergoing programmed population control in a microchemostat. Science.

